# Anticonvulsant Profiles of Certain New 6-Aryl-9-substituted-6,9-diazaspiro-[4.5]decane-8,10-diones and 1-Aryl-4-substituted-1,4-diazaspiro[5.5]undecane-3,5-diones

**DOI:** 10.3390/ijms150916911

**Published:** 2014-09-23

**Authors:** Mohamed N. Aboul-Enein, Aida A. El-Azzouny, Mohamed I. Attia, Yousreya A. Maklad, Mona E. Aboutabl, Fatma Ragab, Walaa H. A. Abd El-Hamid

**Affiliations:** 1 Medicinal and Pharmaceutical Chemistry Department (Medicinal Chemistry Group), Pharmaceutical and Drug Industries Research Division, National Research Centre, Dokki, Giza 12622, Egypt; E-Mails: elazzounyaida@yahoo.com (A.A.E.-A.); mattia@ksu.edu.sa (M.I.A.); 2 Department of Pharmaceutical Chemistry, College of Pharmacy, King Saud University, P. O. Box 2457, Riyadh 11451, Saudi Arabia; 3 Medicinal and Pharmaceutical Chemistry Department (Pharmacology Group) Pharmaceutical and Drug Industries Research Division, National Research Centre, Dokki, Giza 12622, Egypt; E-Mails: yousreya_maklad@yahoo.com (Y.A.M.); monaaboutabl@gmail.com (M.E.A.); 4 Department of Pharmaceutical Chemistry, Faculty of Pharmacy, Cairo University, Cairo 11562, Egypt; E-Mail: fatmarag@hotmail.com; 5 Department of Pharmaceutical Chemistry, Faculty of Pharmacy, Misr University for Science & Technology, 6th of October City 12566, Egypt; E-Mail: dr_walaa_hamada@yahoo.com

**Keywords:** cycloalkanones, Strecker synthesis, alkylation, spiro compounds, tetrazole, anticonvulsant

## Abstract

Synthesis and anticonvulsant potential of certain new 6-aryl-9-substituted-6,9-diazaspiro[4.5]decane-8,10-diones (**6a**–**l**) and 1-aryl-4-substituted-1,4-diazaspiro[5.5]undecane-3,5-diones (**6m**–**x**) are reported. The intermediates 1-[(aryl)(cyanomethyl)amino]cycloalkanecarboxamides (**3a**–**f**) were prepared via adopting Strecker synthesis on the proper cycloalkanone followed by partial hydrolysis of the obtained nitrile functionality and subsequent *N*-cyanomethylation. Compounds **3a**–**f** were subjected to complete nitrile hydrolysis to give the respective carboxylic acid derivatives **4a**–**f** which were cyclized under mild conditions to give the spiro compounds **5a**–**f**. Ultimately, compounds **5a**–**f** were alkylated or aralkylated to give the target compounds **6a**–**i** and **6m**–**u**. On the other hand, compounds **6j**–**l** and **6v**–**x** were synthesized from the intermediates **5a**–**f** through alkylation, dehydration and finally tetrazole ring formation. Anticonvulsant screening of the target compounds **6a**–**x** revealed that compound **6g** showed an ED_50_ of 0.0043 mmol/kg in the scPTZ screen, being about 14 and 214 fold more potent than the reference drugs, Phenobarbital (ED_50_ = 0.06 mmol/kg) and Ethosuximide (ED_50_ = 0.92 mmol/kg), respectively. Compound **6e** exhibited an ED_50_ of 0.019 mmol/kg, being about 1.8 fold more potent than that of the reference drug, Diphenylhydantoin (ED_50_ = 0.034 mmol/kg) in the MES screen. Interestingly, all the test compounds **6a**–**x** did not show any minimal motor impairment at the maximum administered dose in the neurotoxicity screen.

## 1. Introduction

Epilepsy is a group of neurological disorders characterized by excessive abnormal bioelectrical functions of the brain leading to recurrent unprovoked seizures [1,2]. It affects about 1% of the global population with the majority of cases being in the developing countries [3]. Estimates suggest that approximately 20%–30% of patients are not adequately controlled by the available antiepileptic medications [4,5]. Furthermore, the clinically used antiepileptics display serious side effects such as ataxia, hepatotoxicity, gingival hyperplasia and megaloblastic anaemia [6,7,8]. Therefore, there is a substantial need for novel, more effective and more selective antiepileptic agents with lesser side effects.

Diketopiperazines (DKPs) are the smallest cyclic peptides known, commonly biosynthesized from amino acids by a large variety of organisms [9]. They are privileged structures for the discovery of new lead compounds. They display attractive chemical characteristics, such as resistance to proteolysis, mimicking of peptidic pharmacophoric groups, conformational rigidity and donor as well as acceptor groups for hydrogen bonding which might influence interactions with biological targets [10].

DKPs include 2,3-DKPs, 2,5-DKPs and 2,6-DKPs (3-aza-glutarimides). Although various methods and synthetic protocols are reported for the synthesis of 2,6-DKPs, there is a paucity of information on their induced biological profiles, including anticonvulsant, antiviral and anticancer activities [2,11,12,13].

Incorporation of lipophilic moieties in the scaffold of new bioactive chemical entities could improve their anticonvulsant potential. Accordingly, cyclohexane and/or cyclopentane moieties were embedded in the skeleton of the new 2,6-DKP derivatives **6a**–**x** aiming to enhance their anticonvulsant activity. On the other hand, the tetrazole moiety is a bioisostere of carboxylic acid functionality and it is an integrated part in the construction of certain anticonvulsants [14,15]. Therefore, compounds **6j**–**l** and **6v**–**x**, bearing a tetrazole moiety, were synthesized and screened for their anticonvulsant potential.

Our research group has previously reported the synthesis and anticonvulsant activity of certain 1-alkyl-1,4-diazaspiro[4.5]decane and [5.5]undecane-3,5-diones [16] as ring expanded hydantoins which are one of the well known classical families of anticonvulsants. As an extension of this study, we describe herein the synthesis and anticonvulsant profile of certain new 6-aryl-9-substituted-6,9-diazaspiro[4.5]decane-8,10-diones (**6a**–**l**) and 1-aryl-4-substituted-1,4-diazaspiro[5.5]undecane-3,5-diones (**6m**–**x**) aiming to get new anticonvulsant biocandidates.

## 2. Results and Discussion

### 2.1. Chemistry

**Scheme 1:**
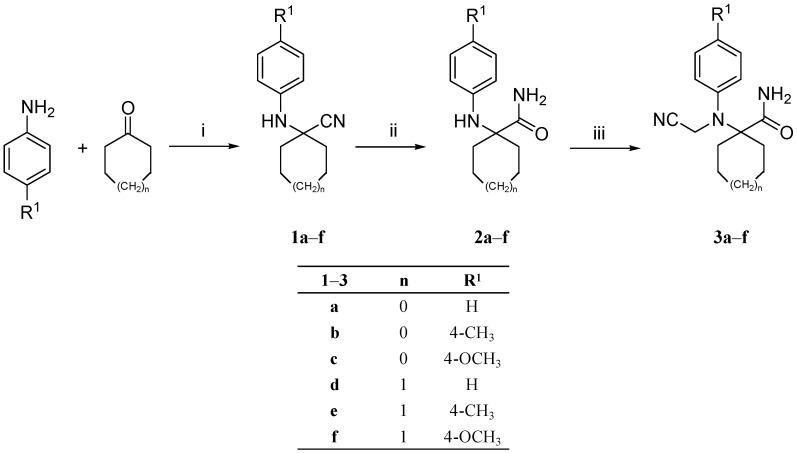
Synthesis of compounds **1**–**3a**–**f**. Reagents and conditions: (i) KCN, glacial acetic acid, RT, 24 h; (ii) Conc. H_2_SO_4_, RT, 48 h; (iii) KCN, formaldehyde 37% solution, paraformaldehyde, 60 °C-RT, 3–18 h.

**Scheme 2:**
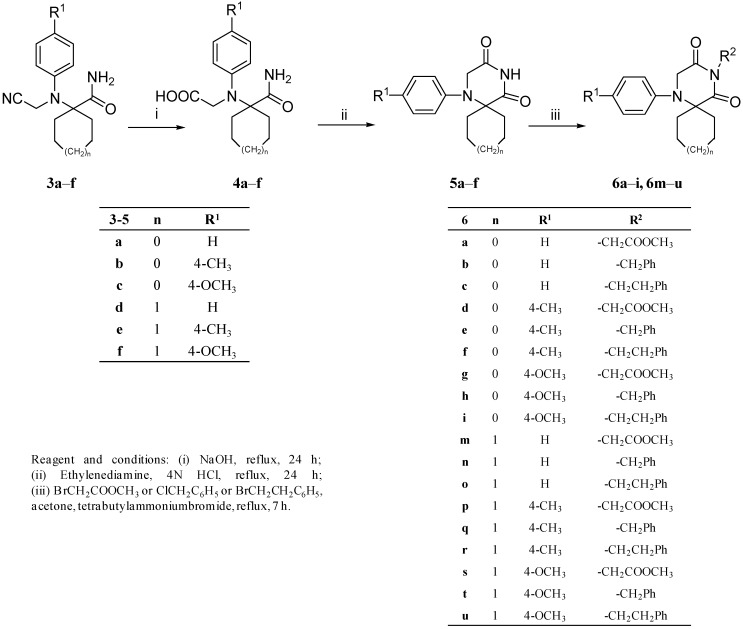
Synthesis of compounds **4a**–**f**, **5a**–**f**, **6a**–**i** and **6m**–**u**.

**Scheme 3:**
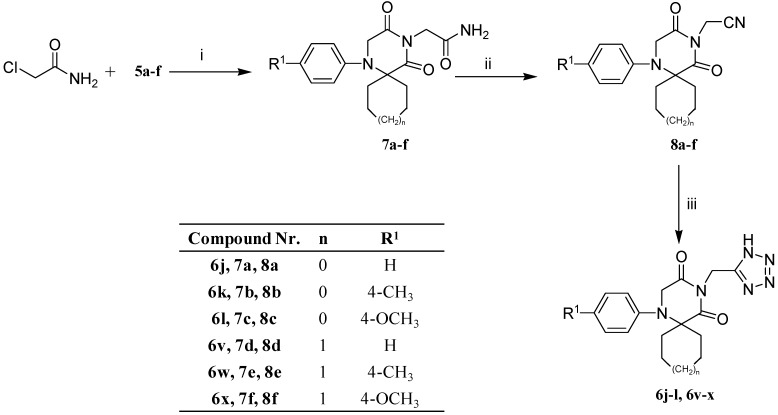
Synthesis of compounds **7a**–**f**, **8a**–**f**, **6j**–**l** and **6v**–**x**. Reagents and conditions: (i) Acetone, K_2_CO_3_, tetrabutylammonium bromide, reflux 7 h; (ii) Triflouroacetic anhydride, THF, cooling, 0–5 °C, 2 h, ammonium bicarbonate; (iii) NaN_3_, AlCl_3_, cooling then reflux 24 h.

Syntheses of the target compounds **6a**–**x** and their intermediates are depicted in Scheme 1, Scheme 2 and Scheme 3. Thus, cyclopentanone and/or cyclohexanone were allowed to react with the appropriate commercially available aniline derivative and potassium cyanide in glacial acetic acid under Strecker synthesis conditions to give the respective nitrile derivatives **1a**–**f**. The nitrile group of compounds **1a**–**f** was subjected to hydrolysis under acidic conditions using sulfuric acid at ambient temperature to yield the amide derivatives **2a**–**f**. Subsequently, cyanomethylation of the secondary amine moiety of compounds **2a**–**f** was successfully achieved using potassium cyanide, paraformaldehyde and formaldehyde to furnish the corresponding compounds **3a**–**f** (Scheme 1).

The target compounds **6a**–**i** and **6m**–**u** as well as their intermediates **4a**–**f** and **5a**–**f** were obtained as portrayed in Scheme 2. Thus, the nitrile moiety in compounds **3a**–**f** was hydrolysed via reflux in sodium hydroxide solution to yield the corresponding carboxylic acid derivatives **4a**–**f**. Cyclization of the latter compounds **4a**–**f** was successfully realized using ethylenediamine in 4 N HCl solution to give the respective spiro compounds **5a**–**f** according to our previously developed procedure [16]. The imide functionality of compounds **5a**–**f** was alkylated under phase transfer catalysis conditions using the appropriate alkyl/aralkyl halide to give the target compounds **6a**–**i** and **6m**–**u**.

The synthesis of the intermediates **7a**–**f** and **8a**–**f** as well as the target compounds **6j**–**l** and **6v**–**x** were successfully achieved as illustrated in Scheme 3. Synthesis of compounds **6j**–**l** and **6v**–**x** was commenced with the reaction of compounds **5a**–**f** with chloroacetamide to give the corresponding compounds **7a**–**f**. Dehydration of compounds **7a**–**f** using trifluoroacetic anhydride furnished the respective penultimate cyanomethyl derivatives **8a**–**f**. Elaboration of the cyano group of compounds **8a**–**f** to the tetrazolyl moiety was acquired using sodium azide in the presence of aluminium chloride to yield the desired compounds **6j**–**l** and **6v**–**x**.

### 2.2. Anticonvulsant Activity

The test compounds **6a**–**x** were subjected to preliminary anticonvulsant evaluation (Phase I screening) according to the protocol given by the Epilepsy Section of the National Institute of Neurological Disorders and Stroke (NINIDS) using the standard procedure adopted by the Antiepileptic Drug Development (ADD) program [17]. Those include the ‘gold standard’ screens, namely subcutaneous Pentylenetetrazole (scPTZ) screen and the maximal electroshock seizure (MES) screen. The former screen identifies compounds that elevate seizure threshold while the latter one measures the ability of the test compound to prevent seizure spread. Compounds exhibited 100% protection against induced seizures, were subjected to median effective dose (ED_50_) estimation and minimal motor impairment (neurotoxicity) evaluation. 

It has been indicated that PTZ-induced seizures can be prevented by drugs that reduce T-type Ca^2+^ currents such as Ethosuximide and also by drugs that enhance gamma amino butyric acid type A (GABA_A_) receptor-mediated inhibitory neurotransmission such as Phenobarbital [18].

The results of the initial anticonvulsant screening of the test compounds **6a**–**x** are given in Table 1. The evaluation indicated that, all the compounds were effective in scPTZ screen while most of them were effective in MES screen. scPTZ screen showed that, compound **6g** (R^1^ = 4-OCH_3_ and R^2^ = -CH_2_COOCH_3_) was the most potent congener in the cyclopentane series **6a**–**l**, displaying 100% protection against PTZ-induced seizure at dose level of 0.0086 mmol/kg as compared with Phenobarbital (0.13 mmol/kg) and Ethosuximide (1.06 mmol) which were used as reference standards.

Meanwhile, compound **6b** (R^1^ = H, R^2^ = CH_2_-Ph) and compound **6d** (R^1^ = 4-CH_3_, R^2^ = -CH_2_COOCH_3_) exerted equal anticonvulsant activity (100% protection) at a dose level of 0.018 mmol/kg. Moreover, all compounds of the cyclopentane series **6a**–**l** were more potent than the reference drugs as they showed the same anti-seizure profile (100% protection) at lower doses on molecular bases (Table 1). The different congeners of this series showed anticonvulsant potential in the following decreasing order:

**6g** > **6b** = **6d** > **6i** > **6a** > **6e** = **6f** > **6k** > **6c** > **6j** > **6l** > **6h**

**Table 1:** Anticonvulsant potential (scPTZ and MES screens) of compounds **6a**–**x** as well as the reference standards, Phenobarbital, Ethosuximide and Diphenylhydantoin in adult male albino mice.

Compound Nr.	Dose (mmol/kg) *	% Protection	
		scPTZ	MES
**6a**	0.0280	100	50
**6b**	0.0180	100	50
**6c**	0.0570	100	60
**6d**	0.0180	100	50
**6e**	0.0320	100	100
**6f**	0.0320	100	60
**6g**	0.0086	100	60
**6h**	0.1300	100	60
**6i**	0.0230	100	33
**6j**	0.0600	100	20
**6k**	0.0350	100	40
**6l**	0.0780	100	0
**6m**	0.0360	100	33
**6n**	0.1400	100	66
**6o**	0.2700	100	50
**6p**	0.1400	100	0
**6q**	0.0690	100	80
**6r**	0.0310	100	0
**6s**	0.0350	100	0
**6t**	0.0310	100	0
**6u**	0.2500	83.3	33
**6v**	0.0470	100	60
**6w**	0.0560	100	40
**6x**	0.0320	100	60
Phenobarbital	0.1300	100	-
Ethosuximide	1.0600	100	-
Diphenylhydantoin	0.1600	-	100

* The minimal dose which exhibits the maximum anticonvulsant activity; The dash (-) indicates the absence of anticonvulsant activity at the tested dose level.

Regarding the cyclohexane series **6m-x**, compounds **6r** (R^1^ = 4-CH_3_, R^2^ = -CH_2_CH_2_Ph) and **6t** (R^1^ = 4-OCH_3_, R^2^ = -CH_2_Ph) exhibited the highest anticonvulsant potential with 100% protection against PTZ-induced seizures in mice at the same dose level of 0.031 mmol/kg. Meanwhile, compound **6o** (R^1^ = H, R^2^ = -CH_2_CH_2_Ph) and compound **6u** (R^1^ = 4-OCH_3_, R^2^ = -CH_2_CH_2_Ph) require high doses to achieve the 100% protection (0.27 and 0.25 mmol/kg, respectively).

The different congeners of the cyclohexane series **6m**–**x** showed a decrease in the anticonvulsant potential in the following decreasing order:

**6r** = **6t** > **6x** > **6s** > **6m** > **6v** > **6w** > **6q** > **6n** = **6p** > **6u** > **6o**

Concerning the MES test, the dose which exerted 100% anticonvulsant protection in the scPTZ screening has been selected. In this screening test, all of the compounds showed protection in half or more of the tested mice after 0.5 h post administration except compounds **6i**, **6j**, **6k**, **6m**, **6u** and **6w**. On the other hand, compounds **6l**, **6p**, **6r**, **6s** and **6t** were devoid from anticonvulsant activity. Meanwhile, **6e** (R^1^ = 4-CH_3_, R^2^ = -CH_2_Ph) exhibited 100% protection at dose level of 0.032 mmol/kg being more potent than the reference drug, Diphenylhydantoin, which exerted the same protection at a dose level of 0.16 mmol/kg. It is worthwhile to mention that, compound **6e** displayed 100% protection against both scPTZ and MES-induced seizures in mice.

Compounds showed 100% protection in scPTZ and/or MES screens, were subjected to median effective dose (ED_50_) estimation as well as to minimal motor impairment (neurotoxicity) evaluation. Table 2 summarizes ED_50_ of the selected test compounds along with their neurotoxicity evaluation. Compound **6g** gave an ED_50_ of 0.0043 mmol/kg ≡ 1.5 mg/kg in the scPTZ screen being about 14 and 214 fold more potent than the reference drugs, Phenobarbital (ED_50_ = 0.06 mmol/kg ≡ 13.2 mg/kg) and Ethosuximide (ED_50_ = 0.92 mmol/kg ≡ 130 mg/kg), respectively. In the MES screen, only compound **6e** showed 100% protection against induced seizures with ED_50_ of 0.019 mmol/kg ≡ 7.0 mg/kg being about 1.8 fold more potent than that of the reference drug, Diphenylhydantoin (ED_50_ = 0.034 mmol/kg ≡ 9.5 mg/kg [19]). Interestingly, all the test compounds did not show any minimal motor impairment at the maximum administered dose in the neurotoxicity screen.

**Table 2:** Median effective dose (ED_50_, mg/kg) of compounds **6a**–**t** and **6v**–**x** exhibiting 100% protection against scPTZ-induced seizers and their neurotoxicity in adult male albino mice using Phenobarbital and Ethosuximide as reference standards.

Compound Nr.	ED_50_ (Confidence Limits)	Neurotoxicity *
**6a**	4.5 (6.85–2.96)	0/6
**6b**	2.5 (3.56–1.76)	0/6
**6c**	11.5 (13.66–9.68)	0/6
**6d**	2.4 (4.10–1.40)	0/6
6e **	6.0 (7.95–4.53)	0/6
**6f**	2.5 (2.84–2.20)	0/6
**6g**	1.5 (2.40–0.94)	0/6
**6h**	19.0 (25.24–14.30)	0/6
**6i**	4.2 (6.82–2.59)	0/6
**6j**	13.5 (15.38–11.85)	0/6
**6k**	6.5 (8.44–4.27)	0/6
**6l**	16.5 (19.65–13.85)	0/6
**6m**	4.0 (6.92–2.31)	0/6
**6n**	25.0 (33.35–18.74)	0/6
**6o**	35.0 (66.42–18.44)	0/6
**6p**	24.0 (32.24–17.87)	0/6
**6q**	10.0 (13.67–7.32)	0/6
**6r**	4.0 (6.14–2.60)	0/6
**6s**	6.0 (8.79–4.09)	0/6
**6t**	6.0 (8.44–4.27)	0/6
**6v**	6.5 (11.89–3.55)	0/6
**6w**	12.0 (15.42–9.34)	0/6
**6x**	6.0 (11.00–3.27)	0/6
Phenobarbital	13.2 (15.90–6.80)	ND
Ethosuximide	130.0 (111–150)	ND

* Rotarod test: number of animals exhibiting neurotoxicity/number of animals tested; ** ED_50_ in MES screen = 7.0 mg/kg; ND: not determined.

## 3. Experimental Section

### 3.1. Chemistry

All melting points were determined using Electrothermal Capillary melting point apparatus and are uncorrected. Infrared (IR) spectra were recorded as thin film (for oils) in NaCl discs or as KBr pellets (for solids) with JASCO FT/IR-6100 spectrometer and values are represented in cm^−^^1^. ^1^H-NMR (500 MHz) and ^13^C-NMR (125 MHz) spectra were carried out on Jeol ECA 500 MHz spectrometer using TMS as internal standard and chemical shift values were recorded in ppm on δ scale. The ^1^H-NMR data were represented as follows: chemical shifts, multiplicity (s. singlet, d. doublet, t. triplet, m. multiplet, br. broad), number of protons, and type of protons. The ^13^C-NMR data were represented as chemical shifts and type of carbons. Mass spectral data were obtained with electron impact (EI) ionization technique at 70 eV from a Finnigan Mat SSQ-7000 Spectrometer. Elemental analyses were carried out in Microanalytical Units at National Research Centre and Cairo University. Silica gel TLC (thin layer chromatography) cards from Merck (silica gel precoated aluminum cards with fluorescent indicator at 254 nm) were used for thin layer chromatography. Visualization was performed by illumination with UV light source (254 nm). Column chromatography was carried out on silica gel 60 (0.063–0.200 mm) obtained from Merck.

#### 3.1.1. General Procedure for the Synthesis of 1-(Arylamino)cycloalkanecarbonitriles (**1a**–**f**)

A solution of potassium cyanide (9.75 g, 0.15 mol) in water (25 mL) was added drop-wise to a solution of cycloalkanone (0.15 mol) and the appropriate aniline derivative (0.15 mol) in glacial acetic acid (75 mL). The reaction mixture was stirred mechanically at room temperature for 24 h. The precipitated product was filtered off, washed with water, dried and recrystallized from petroleum ether (40–60 °C) to afford **1a**–**f**. The spectral data of compounds **1a**–**f** were consistent with the published ones (cited below).

1-(Phenylamino)cyclopentanecarbonitrile (**1a**) [20]. Yield: 92%; white solid, m.p. 60 °C.

1-[(4-Methylphenyl)amino]cyclopentanecarbonitrile (**1b**) [21]. Yield: 89%; buff solid, m.p. 56 °C.

1-[(4-Methoxyphenyl)amino]cyclopentanecarbonitrile (**1c**) [22]. Yield: 80%; brown solid, m.p. 132 °C.

1-(Phenylamino)cyclohexanecarbonitrile (**1d**) [23]. Yield: 75%; white solid, m.p. 74–76 °C.

1-[(4-Methylphenyl)amino]cyclohexanecarbonitrile (**1e**) [24]. Yield: 75%; yellowish white solid, m.p. 76–78 °C.

1-[(4-Methoxyphenyl)amino]cyclohexanecarbonitrile (**1f**) [24]. Yield: 84.5%; buff solid, m.p. 76 °C.

#### 3.1.2. General Procedure for the Synthesis of 1-(Arylamino)cycloakanecarboxamides (**2a**–**f**)

The appropriate nitrile derivative **1a**–**f** (0.125 mol) was dissolved in cold concentrated sulfuric acid (20 mL). After remaining at room temperature for 48 h, the reaction mixture was poured over crushed ice and rendered alkaline with 25% ammonium hydroxide solution. The precipitated amide was filtered off, washed with water, dried and recrystallized from ethanol to give **2a**–**f**. The spectral data of compounds **2a**–**f** were consistent with the published ones (cited below).

1-(Phenylamino)cyclopentanecarboxamide (**2a**) [25]. Yield: 90%; white solid, m.p. 166 °C.

1-[(4-Methylphenyl)amino]cyclopentanecarboxamide (**2b**) [26]. Yield: 85%; buff solid, m.p. 120 °C.

1-[(4-Methoxyphenyl)amino]cyclopentanecarboxamide (**2c**) [27]. Yield: 50%; buff solid, m.p. 90–93 °C.

1-(Phenylamino)cyclohexanecarboxamide (**2d**) [24]. Yield: 85%; white solid, m.p. 148 °C.

1-[(4-Methylphenyl)amino]cyclohexanecarboxamide (**2e**) [27]. Yield: 85%; white solid, m.p. 154 °C.

1-[(4-Methoxyphenyl)amino]cyclohexanecarboxamide (**2f**) [27]. Yield: 75%; buff solid, m.p. 110 °C.

#### 3.1.3. General Procedure for the Synthesis of 1-[(Aryl)(cyanomethyl)amino]cycloalkanecarboxamides (**3a**–**f**)

Paraformaldehyde (1.52 g, 0.05 mol) was added to a solution of the appropriate 1-(arylamino)cycloakanecarboxamides (**2a**–**f**) (0.05 mol) in glacial acetic acid (30 mL). A solution of potassium cyanide (3.9 g, 0.06 mol) was added drop-wise to the stirred and cooled (15 °C) reaction mixture. The temperature was raised gradually to 45 °C over 30 min and was maintained at 50–60 °C for 3 h. After cooling to 35 °C, a 37% formaldehyde solution (5 mL) was added and the reaction mixture was stirred at room temperature for 18 h. Water (30 mL) was added, the reaction mixture was cooled and neutralized with 10% sodium carbonate solution. The precipitated product was extracted with CH_2_Cl_2_ (3 × 50 mL), washed with water (2 × 30 mL), dried (Na_2_SO_4_) and evaporated under vacuum to give the anticipated compounds **3a**–**f**. The crude **3a**–**f** were pure enough to be used in the following step without any further purification. The spectral data of compounds **3a**–**f** were consistent with the published ones (cited below).

1-[(Cyanomethyl)(phenyl)amino]cyclopentanecarboxamide (**3a**) [16]. Yield: 78%; pale yellow viscous oil.

1-[(Cyanomethyl)(4-methylphenyl)amino]cyclopentanecarboxamide (**3b**) [16]. Yield: 86.6%; pale yellow viscous oil.

1-[(Cyanomethyl)(4-methoxyphenyl)amino]cyclopentanecarboxamide (**3c**) [16]. Yield: 80%; pale yellow viscous oil.

1-[(Cyanomethyl)(phenyl)amino]cyclohexanecarboxamide (**3d**) [16]. Yield: 85%; yellowish white solid, m.p. 135 °C.

1-[(Cyanomethyl)(4-methylphenyl)amino]cyclohexanecarboxamide (**3e**) [16]. Yield: 95%; buff solid, m.p. 83 °C.

1-[(Cyanomethyl)(4-methoxyphenyl)amino]cyclohexanecarboxamide (**3f**) [16]. Yield: 97%; buff solid, m.p. 103 °C.

#### 3.1.4. General Procedure for the Synthesis of [(Aryl)(1-carbamoylcycloalkyl)amino]acetic Acids (**4a**–**f**)

A mixture of the appropriate cyanomethyl derivative **3a**–**f** (0.01 mol) and NaOH (0.48 g, 0.012 mol) in 50% aqueous ethanol (25 mL) was stirred under reflux for 18 h, utill complete evolution of ammonia was ceased. The ethanol was removed by evaporation under vacuum. The residue was extracted with ethyl acetate (2 × 15 mL) and the aqueous layer was acidified with 2 N HCl. The acidic layer was extracted with ethyl acetate (3 × 15 mL), dried (Na_2_SO_4_) and evaporated under reduced pressure to yield compounds **4a**–**f**. The crude **4a**–**f** were pure enough to be used in the following step without any further purification. The spectral data of compounds **4a**–**f** were consistent with the published ones (cited below).

[(1-Carbamoylcyclopentyl)(phenyl)amino]acetic acid (**4a**) [16]. Yield: 85%; white solid, m.p. 120–121 °C.

[(1-Carbamoylcyclopentyl)(4-methylphenyl)amino]acetic acid (**4b**) [16]. Yield: 80%; yellowish white solid, m.p. 118 °C.

[(1-Carbamoylcyclopentyl)(4-methoxyphenyl)amino]acetic acid (**4c**) [16]. Yield: 70%; buff solid, m.p. 105 °C.

[(1-Carbamoylcyclohexyl)(phenyl)amino]acetic acid (**4d**) [16]. Yield: 70%; white solid, m.p. 186 °C.

[(1-Carbamoylcyclohexyl)(4-methylphenyl)amino]acetic acid (**4e**) [16]. Yield: 80%; buff solid, m.p. 188 °C.

[(1-Carbamoylcyclohexyl)(4-methoxyphenyl)amino]acetic acid (**4f**) [16]. Yield: 70%; buff solid, m.p. 163 °C.

#### 3.1.5. General Procedure for the Synthesis of 6-Aryl-6,9-diazaspiro-[4.5]decane-8,10-diones (**5a**–**c**) and 1-Aryl-1,4-diazaspiro[5.5]undecane-3,5-diones (**5d**–**f**)

4 N HCl (40 mL, 0.16 mol) was added to a solution of the appropriate carboxylic acid derivative **4a**–**f** (0.01 mol) and ethylenediamine (3.61 g, 0.06 mol) in dioxan (60 mL). The reaction mixture was refluxed under stirring for 18 h. The solvent was evaporated *in vacuo* and the residue was neutralized (pH 6–7) with 5% NaHCO_3_ solution utill no effervescence occured, extracted with CH_2_Cl_2_ (3 × 20 mL), dried (Na_2_SO_4_) and the organic layer was evaporated under reduced pressure to give compounds **5a**–**f**. The crude **5a**–**f** were pure enough to be used in the following step without any further purification. The spectral data of compounds **5a**–**f** were consistent with the published ones (cited below).

6-Phenyl-6,9-diazaspiro[4.5]decane-8,10-dione (**5a**) [16]. Yield: 50%; white solid, m.p. 73–74 °C.

6-(4-Methylphenyl)-6,9-diazaspiro[4.5]decane-8,10-dione (**5b**) [16]. Yield: 60%; white solid, m.p. 88 °C.

6-(4-Methoxyphenyl)-6,9-diazaspiro[4.5]decane-8,10-dione (**5c**) [16]. Yield: 50%; buff solid m.p. 60 °C.

1-Phenyl-1,4-diazaspiro[5.5]undecane-3,5-dione (**5d**) [16]. Yield: 80%; white solid, m.p. 162 °C.

1-(4-Methylphenyl)-1,4-diazaspiro[5.5]undecane-3,5-dione (**5e**) [16]. Yield: 85%; white solid, m.p. 183 °C.

1-(4-Methoxyphenyl)-1,4-diazaspiro[5.5]undecane-3,5-dione (**5f**) [16]. Yield: 60%; buff solid, m.p. 110 °C.

#### 3.1.6. General Procedure for the Synthesis of 6-Aryl-9-substituted-6,9-diazaspiro-[4.5]decane-8,10-diones (**6a**–**i**) and 1-Aryl-4-substituted-1,4-diazaspiro[5.5]undecane-3,5-diones (**6m**–**u**)

To a mixture of the appropriate diketopiperazine derivative **5a**–**f** (0.01 mol) in acetone (100 mL), was added the proper alkylating agent (0.07 mol), namely methyl bromoacetate, benzyl chloride or phenethylbromide in the presence of K_2_CO_3_ (1.38 g, 0.01 mol) and a catalytic amount of tetrabutylammoniun bromide (0.32 g, 0.001 mol) as a phase transfer catalyst. The reaction mixture was heated under reflux for 7 h, cooled to room temperature, filtered and the filtrate was evaporated under vacuum. The residue was purified using column chromatography (chloroform:ethyl acetate, 9:1) to furnish the target compounds **6a**–**i** and **6m**–**u**.

Methyl 2-(8,10-dioxo-6-phenyl-6,9-diazaspiro[4.5]decane-9-yl)acetate (**6a**). Yield: 65%; yellow viscous oil; IR (KBr, *ν*, cm^−1^) absence of NH band at 3100 and exhibited bands at 1752 (carbonyl ester), 1720, 1685 (imide carbonyls), 609, 557; ^1^H-NMR (CDCl_3_) δ ppm 1.80 (br.s, 4H, 2 × CH_2_, cyclopentyl), 2.00–2.37 (m, 4H, 2 × CH_2_, cyclopentyl), 3.76 (s, 3H, COOCH_3_), 4.32 (s, 2H, O=C-CH_2_-N), 4.58 (s, 2H, N-CH_2_-COO), 7.02–7.32 (m, 5H, H_ar_.); ^13^C-NMR (CDCl_3_) δ ppm 25.08, 36.25 (4 × CH_2_, cyclopentyl), 40.12 (CH_2_-COOCH_3_), 52.59, 56.59 (O=C-CH_2_-N, COOCH_3_), 69.65 (Cq), 124.82, 128.48, 129.32 (CH_ar_.), 148.71 (C_ar_.), 169.91, 170.11, 176.18 (3 × C=O); MS (EI) *m*/*z* (%): 316.2 ([M]^+^, 17), 91 (100), 172.2 (90); Anal. Calcd for C_17_H_20_N_2_O_4_ (316.35): C, 64.54%; H, 6.37%; N, 8.86%. Found: C, 64.51%; H, 6.15%; N, 8.66%.

9-Benzyl-6-phenyl-6,9-diazaspiro[4.5]decane-8,10-dione (**6b**). Yield: 60%; Yellow viscous oil; IR (KBr, *ν*, cm^−1^) absence of NH band at 3100 and exhibited bands at 1725, 1678 (imide carbonyls), 604, 555; ^1^H-NMR (CDCl_3_) δ ppm 1.79–2.30 (m, 8H, 4 × CH_2_, cyclopentyl), 4.27 (s, 2H, O=C-CH_2_-N), 5.10 (s, 2H, CH_2_-C_6_H_5_), 6.85–6.86 (m, 2H, H_ar_.), 7.05–7.17 (m, 3H, H_ar_.), 7.30–7.34 (m, 5H, H_ar_.); ^13^C-NMR (CDCl_3_) δ ppm 25.50, 36.70 (4 × CH_2_, cyclopentyl), 42.6 (CH_2_-C_6_H_5_), 56.91 (O=C-CH_2_-N), 67.27 (Cq), 124.71, 126.96, 127.49, 128.21, 128.40, 129.42 (CH_ar_.), 136.81, 148.76 (2 × C_ar_.), 170.33, 176.00 (2 × C=O); MS (EI) *m*/*z* (%): 334.3 ([M]^+^, 15), 91 (100), 77.1 (40); Anal. Calcd. for C_21_H_22_N_2_O_2_ (334.41): C, 75.42%; H, 6.63%; N, 8.38%. Found: C, 75.32%; H, 6.61%; N, 8.17%.

9-Phenethyl-6-phenyl-6,9-diazaspiro[4.5]decane-8,10-dione (**6c**). Yield: 71.5%; yellow viscous oil; IR (KBr, *ν*, cm^−1^) absence of NH band at 3100 and exhibited bands at 1725, 1677 (carbonyl imides), 575, 500; ^1^H-NMR (CDCl_3_) δ ppm 1.77 (br.s, 4H, 2 × CH_2_, cyclopentyl), 1.96 (s, 2H, CH_2_, cyclopentyl), 2.25 (s, 2H, CH_2_, cyclopentyl), 2.85 (t, 2H, *J* = 7.5 Hz, CH_2_-C_6_H_5_) , 4.09 (t, 2H, *J* = 7.5 Hz, CH_2_-CH_2_-C_6_H_5_), 4.24 (s, 2H, O=C-CH_2_-N), 6.95–7.12 (m, 3H, H_ar_.), 7.26–7.32 (m, 7H, H_ar_.); ^13^C-NMR (CDCl_3_) δ ppm 25.05, 33.92 (4 × CH_2_, cyclopentyl), 36.59, 40.53 (CH_2_-C_6_H_5_, CH_2_-CH_2_-C_6_H_5_), 56.92 (O=C-CH_2_-N), 63.71 (Cq), 124.53, 124.62, 126.52, 128.45, 128.61, 129.05 (CH_ar_.), 138.27, 148.82 (2 × C_ar_.), 170.03, 175.97 (2 × C=O); MS (EI) *m*/*z* (%): 348.23 ([M]^+^, 22), 91 (100), 172.1 (65), 229 (65), Anal. Calcd. for C_22_H_24_N_2_O_2_ (348.44): C, 75.38%; H, 6.94%; N, 8.04%. Found: C, 75.41%; H, 6.91%; N, 8.23%.

Methyl 2-(8,10-dioxo-6-(4-methylphenyl)-6,9-diazaspiro[4.5]decane-9-yl)acetate (**6d**). Yield: 79%; yellow viscous oil; IR (KBr, *ν*, cm^−1^) absence of NH band at 3100 and exhibited bands at 1752 (carbonyl ester), 1722, 1686 (imide carbonyls), 607, 564; ^1^H-NMR (CDCl_3_) δ ppm 1.73–1.77 (m, 8H, 4 × CH_2_, cyclopentyl), 2.29 (s, 3H, CH_3_), 3.75 (s, 3H, COOCH_3_), 4.23 (s, 2H, O=C-CH_2_-N), 4.65 (s, 2H, CH_2_-COOCH_3_), 6.91 (d, 2H, *J* = 8.6 Hz, H_ar_), 7.05 (d, 2H, *J* = 8.6 Hz, H_ar_.); ^13^C-NMR (CDCl_3_) δ ppm 21.31 (CH_3_), 25.62, 36.85 (4 × CH_2_, cyclopentyl), 40.16 (CH_2_-COOCH_3_), 52.53, 56.89 (O=C-CH_2_-N, COOCH_3_), 69.81 (Cq), 124.90, 130.57, (CH_ar_.), 134.56, 146.22 (2 × C_ar_.), 170.17, 176.01 (2 × C=O); MS (EI) *m*/*z* (%): 330.24 ([M]^+^, 24), 105.1(100), 186.2 (53); Anal. Calcd. for C_18_H_22_N_2_O_4_ (330.38): C, 65.44%; H, 6.71%; N, 8.48%. Found: C, 65.21%; H, 6.63%; N, 8.38%.

9-Benzyl-6-(4-methylphenyl))-6,9-diazaspiro[4.5]decane-8,10-dione (**6e**). Yield: 90%; colourless viscous oil; IR (KBr, *ν*, cm^−1^) absence of NH band at 3100 and exhibited bands at 1725, 1678 (imide carbonyls), 634, 582; ^1^H-NMR (CDCl_3_) δ ppm 1.75–1.91 (m, 8H, 4 × CH_2_, cyclopentyl), 2.23 (s, 3H, CH_3_), 4.20 (s, 2H, O=C-CH_2_-N), 5.01 (s, 2H, CH_2_-C_6_H_5_), 6.71 (d, 2H, *J* = 8.6 Hz, H_ar_.), 7.25 (d, 2H, *J* = 8.6 Hz, H_ar_.), 7.27–7.33 (m, 5H, H_ar_.); ^13^C-NMR (CDCl_3_) δ ppm 20.85 (CH_3_), 25.12, 36.71 (4 × CH_2_, cyclopentyl), 42.72 (CH_2_-C_6_H_5_), 56.89 (O=C-CH_2_-N), 69.97 (Cq), 124.98, 127.57, 128.50, 128.94, 129.76 (CH_ar_.), 134.57, 136.92, 146.27 (3 × C_ar_.), 170.59, 176.19 (2 × C=O); MS (EI) *m*/*z* (%): 364.26 ([M]^+^, 28), 91(100), 105 (98); Anal. Calcd. for C_22_H_24_N_2_O_3_ (364.44): C, 72.50%; H, 6.64%; N, 7.69%. Found: C, 72.43%; H, 6.75%; N, 7.81%.

6-(4-Methylphenyl)-9-phenethyl-6,9-diazaspiro[4.5]decane-8,10-dione (**6f**). Yield: 71.2%; yellow viscous oil; IR (KBr, *ν*, cm^−1^) absence of NH band at 3100 and exhibited bands at 1725, 1678 (imide carbonyls), 646, 606; ^1^H-NMR (CDCl_3_) δ ppm 1.73–2.17 (m, 8H, 4 × CH_2_, cyclopentyl), 2.20–2.27 (m, 3H, CH_3_), 2.84 (t, *J* = 7.7 Hz, 2H, CH_2_-C_6_H_5_), 4.04 (t, 2H, *J* = 7.7 Hz, CH_2_-CH_2_), 4.16 (s, 2H, O=C-CH_2_-N), 7.21 (d, 2H, *J* = 6.7 Hz, H_ar_.), 7.24 (d, 2H, *J* = 6.7 Hz, H_ar_.), 7.25–7.26 (m, 5H, H_ar_.); ^13^C-NMR (CDCl_3_) δ ppm 20.83 (CH_3_), 25.09, 34.08, (4 × CH_2_, cyclopentyl), 36.59, 40.58 (CH_2_-C_6_H_5_, CH_2_-CH_2_-C_6_H_5_), 56.90 (O=C-CH_2_-N), 69.88 (Cq), 124.63, 126.56, 128.68, 128.68, 129.13 (CH_ar_.), 134.40, 138.39, 146.30 (3 × C_ar_.), 170.30, 176.18 (2 × C=O); MS (EI) *m*/*z* (%): 362.2 ([M]^+^, 15), 81(100); Anal. Calcd. for C_23_H_26_N_2_O_2_ (362.46): C, 76.21%; H, 7.23%; N, 7.73%. Found: C, 76.02%; H, 7.15%; N, 7.89%.

Methyl 2-(6-(4-methoxyphenyl)-8,10-dioxo-6,9-diazaspiro[4.5]decan-9-yl)acetate (**6g**). Yield: 71.4%; yellow viscous oil; IR (KBr, *ν*, cm^−1^) absence of NH band at 3100 and exhibited bands 1752 (carbonyl ester), 1720, 1685 (imide carbonyls), 609, 557; ^1^H-NMR (CDCl_3_) δ ppm 1.79–1.82 (m, 4H, 2 × CH_2_, cyclopentyl), 2.25–2.28 (m, 4H, 2 × CH_2_, cyclopentyl), 3.82 (s, 6H, COOCH_3_, OCH_3_), 4.25 (s, 2H, O=C-CH_2_-N), 4.61 (s, 2H, CH_2_-COOCH_3_), 6.83 (d, 2H, *J* = 9.0 Hz, H_ar_.), 7.12 (d, 2H, *J* = 9.0 Hz, H_ar_.); ^13^C-NMR (CDCl_3_) δ ppm 24.95, 36.24 (4 × CH_2_, cyclopentyl), 40.19 (CH_2_-COOCH_3_), 51.97, 52.44, 55.45 (O=C-CH_2_-N, COOCH_3_, OCH_3_), 70.68 (Cq), 114.40, 114.99 (CH_ar_.), 133.40, 157.42, (2 × C_ar_.), 169.69, 170.59, 176.19 (3 × C=O); MS (EI) *m*/*z* (%): 346.23 ([M]^+^, 17), 121.14 (100), 77.1 (29); Anal. Calcd. for C_18_H_22_N_2_O_5_ (346.38): C, 62.42%; H, 6.40%; N, 8.09%. Found: C, 62.22%; H, 6.35%; N, 8.22%.

9-Benzyl-6-(4-methoxyphenyl)-6,9-diazaspiro[4.5]decane-8,10-dione (**6h**). Yield: 90%; yellow viscous oil; IR (KBr, *ν*, cm^−1^) absence of NH band at 3100 and exhibited bands at 1725, 1678 (imide carbonyls), 634, 582; ^1^H-NMR (CDCl_3_) δ ppm 1.78 (br.s, 4H, 2 × CH_2_, cyclopentyl), 1.92–2.21 (m, 4H, 2 × CH_2_, cyclopentyl), 3.78 (s, 3H, OCH_3_), 4.17 (s, 2H, O=C-CH_2_-N), 5.51 (s, 2H, CH_2_-C_6_H_5_), 6.67 (d, 2H, *J* = 8.5 Hz, H_ar_.), 6.75 (d, 2H, *J* = 8.5 Hz, H_ar_.), 7.28–7.39 (m, 5H, H_ar_.); ^13^C-NMR (CDCl_3_) δ ppm 25.58, 36.40 (4 × CH_2_, cyclopentyl), 39.0 (CH_2_-C_6_H_5_), 42.63 (O=C-CH_2_-N), 55.48 (OCH_3_), 73.15 (Cq), 114.27, 114.96, 126.50, 127.54, 128.18 (CH_ar_.), 135.47, 136.78, 141.35 (3 × C_ar_.), 170.43, 172.47 (2 × C=O); MS (EI) *m*/*z* (%): 364.2 ([M]^+^, 28), 91(100), 121 (85); Anal. Calcd. for C_22_H_24_N_2_O_3_ (364.44): C, 72.50%; H, 6.64%; N, 7.96%. Found: C, 72.33%; H, 6.46%; N, 7.79%.

6-(4-Methoxyphenyl)-9-phenethyl-6,9-diazaspiro[4.5]decane-8,10-dione (**6i**). Yield: 90%; yellow viscous oil; IR (KBr, *ν*, cm^−1^) absence of NH band at 3100 and exhibited bands 1722, 1687 (imide carbonyls), 634, 582; ^1^H-NMR (CDCl_3_) δ ppm 1.64–1.83 (m, 8H, 4 × CH_2_, cyclopentyl), 2.79 (t, 3H, *J* = 8.0 Hz, CH_2_-C*H_2_*-C_6_H_5_)), 3.68 (s, 2H, C*H_2_*-CH_2_-C_6_H_5_), 3.99 (s, 3H, OCH_3_), 4.02 (s, 2H, O=C-CH_2_-N), 6.68 (d, 2H, *J* = 9.0 Hz, H_ar_.), 6.79 (d, 2H, *J* = 9.0 Hz, H_ar_.), 7.28–7.39 (m, 5H, H_ar_.); ^13^C-NMR (CDCl_3_) δ ppm 24.94, 36.40 (4 × CH_2_, cyclopentyl), 39.08, 42.63 (CH_2_-C_6_H_5_, O=C-CH_2_-N), 44.75 (CH_2_-CH_2_-C_6_H_5_), 55.41 (OCH_3_), 73.15 (Cq), 114.27, 114.96, 126.50, 128.17, 129.00 (CH_ar._), 135.47, 136.78, 141.35 (3 × C_ar_.), 170.43, 172.47 (2 × C=O); MS (EI) *m*/*z* (%): 378.24 ([M]^+^, 40), 121 (100); Anal. Calcd. for C_23_H_26_N_2_O_3_ (378.46): C, 72.99%; H, 6.92%; N, 7.40%. Found: C, 72.66%; H, 6.99%; N, 7.58%.

Methyl 2-(3,5-dioxo-1-phenyl-1,4-diazaspiro[5.5]undecane-4-yl)acetate (**6m**). Yield: 78%; white solid m.p. 118 °C; IR (KBr, *ν*, cm^−1^) absence of NH band at 3100 and exhibited bands at 1760 (carbonyl ester), 1726, 1675 (imide carbonyls), 633, 588; ^1^H-NMR (CDCl_3_) δ ppm 1.41–2.00 (m, 10H, 5 × CH_2_, cyclohexyl), 3.72 (s, 3H, COOCH_3_), 4.11 (s, 2H, O=C-CH_2_-N), 4.56 (s, 2H, CH_2_-COOCH_3_), 7.12–7.25 (m, 5H, CH_ar_.); ^13^C-NMR (CDCl_3_) δ ppm 20.46, 25.52, 31.48 (5 × CH_2_, cyclohexyl), 40.19 (CH_2_-COOCH_3_), 59.11 (O=C-CH_2_-N), 60.59 (Cq), 126.01, 127.28, 129.36 (CH_ar_.), 147.95 (C_ar_.), 168.46, 170.56, 176.27 (3 × C=O); MS (EI) *m*/*z* (%): 330.1 ([M]^+^, 80), 186.2 (100), 91.1 (49); Anal. Calcd. for C_18_H_22_N_2_O_4_: C, 65.44%; H, 6.71%; N, 8.48%. Found: C, 65.52%; H, 6.68%; N, 8.31%.

4-Benzyl-1-phenyl-1,4-diazaspiro[5.5]undecane-3,5-dione (**6n**). Yield: 80%; colourless viscous oil; IR (KBr, *ν*, cm^−1^) absence of NH band at 3100 and exhibited bands at 1725, 1678 (imide carbonyls), 634, 582; ^1^H-NMR (CDCl_3_) δ ppm 1.48–1.97 (m, 10H, 5 × CH_2_, cyclohexyl), 4.11 (s, 2H, O=C-CH_2_-N), 5.03 (s, 2H, CH_2_-C_6_H_5_), 6.86 (s, 2H, CH_ar_.), 6.86–7.13 (m, 5H, CH_ar_.), 7.38–7.39 (m, 5H, CH_ar_.); ^13^C-NMR (CDCl_3_) δ ppm 20.53, 25.63, 31.57 (5 × CH_2_, cyclohexyl)), 42.66, 55.37 (CH_2_-C_6_H_5_, O=C-CH_2_-N), 60.71 (Cq), 125.84, 127.08, 128.52, 129.17, 129.31, 129.76 (CH_ar_.), 136.94, 148.06 (2 × C_ar_.), 170.94, 176.42 (2 × C=O); MS (EI) *m*/*z* (%): 348.2 ([M]^+^, 100), 186.2 (70), 91.1 (58); Anal. Calcd. for C_22_H_24_N_2_O_2_ (348.44): C, 75.83%; H, 6.94%; N, 8.04%. Found: C, 75.78%; H, 6.88%; N, 8.27%.

4-Phenethyl-1-phenyl-1,4-diazaspiro[5.5]undecane-3,5-dione (**6o**). Yield: 64.5%; buff solid, m.p. 138 °C; IR (KBr, *ν*, cm^−1^) absence of NH band at 3100 and exhibited bands at 1720, 1681 (imide carbonyls), 592, 555; ^1^H-NMR (CDCl_3_) δ ppm 1.48–1.96 (m, 10H, 5 × CH_2_, cyclohexyl), 2.87 (t, 2H, *J* = 7.6 Hz, CH_2_-C_6_H_5_), 4.08 (s, 2H, C*H_2_*CH_2_-C_6_H_5_), 4.12 (s, 2H, O=C-CH_2_-N), 6.99–7.00 (m, 2H, H_ar_.), 7.23–7.29 (m, 8H, H_ar_.); ^13^C-NMR (CDCl_3_) δ ppm 20.57, 25.63, 31.56 (5 × CH_2_, cyclohexyl), 34.12, 40.61 (CH_2_-C_6_H_5_, CH_2_-CH_2_-C_6_H_5_), 55.36 (O=C-CH_2_-N), 60.48 (Cq), 125.78, 126.61, 126.92, 128.56, 129.12, 129.40 (CH_ar_.), 138.39, 148.20 (2 × C_ar_.), 170.74, 176.56 (2 × C=O); MS (EI) *m*/*z* (%): 362.2 ([M]^+^, 100), 243.1 (90), 186.1 (70), 91.1 (48); Anal. Calcd. for C_23_H_26_N_2_O_2_ (362.46): C, 76.21%; H, 7.23%; N, 7.73%. Found: C, 76.41%; H, 7.42%; N, 7.91%. 

Methyl 2-(3,5-dioxo-1-(4-methylphenyl)-1,4-diazaspiro[5.5]undecan-4-yl)acetate (**6p**). Yield: 75%; white solid, m.p. 156–158 °C; IR (KBr, *ν*, cm^−1^) absence of NH band at 3100 and exhibited bands at 1752 (carbonyl ester), 1726, 1675 (imide carbonyls), 619, 523; ^1^H-NMR (CDCl_3_) δ ppm 1.49–1.98 (m, 10H, 5 × CH_2_, cyclohexyl), 2.28 (s, 3H, CH_3_), 3.77 (s, 3H, COOCH_3_), 3.79 (s, 2H, O=C-CH_2_-N), 4.58 (s, 2H, CH_2_-COOCH_3_), 7.07 (s, 4H, H_ar_.); ^13^C-NMR (CDCl_3_) δ ppm 20.94, 21.15, 31.53 (5 × CH_2_, cyclohexyl), 24.35 (CH_3_), 40.19 (CH_2_-COOCH_3_), 52.48, 54.98 (O=C-CH_2_-N, CH_2_COOCH_3_), 60.79 (Cq), 127.08, 129.99 (CH_ar_.), 135.81, 145.32 (2 × C_ar_.), 168.48, 170.64, 176.30 (3 × C=O); MS (EI) *m*/*z* (%): 344.2 ([M]^+^, 50), 200.1 (100), 105(37), 91.1(29); Anal. Calcd. for C_19_H_24_N_2_O_4_ (344.4): C, 66.26%; H, 7.02%; N, 8.13%. Found: C, 66.17%; H, 7.25%; N, 8.31%.

4-Benzyl-1-(4-methylphenyl)-1,4-diazaspiro[5.5]undecane-3,5-dione (**6q**). Yield: 70%; white solid m.p. 110 °C; IR (KBr, *ν*, cm^−1^) absence of NH band at 3100 and exhibited bands at 1717, 1673 (imide carbonyls), 615, 516; ^1^H-NMR (CDCl_3_) δ ppm 1.47–1.87 (m, 10H, 5 × CH_2_, cyclohexyl), 1.92 (s, 3H, CH_3_), 4.07 (s, 2H, O=C-CH_2_-N), 5.07 (s, 2H, CH_2_-C_6_H_5_), 6.92 (d, 2H, *J* = 7.5 Hz, H_ar_.), 7.30 (d, 2H, *J* = 7.5 Hz, H_ar_.), 7.38-7.40 (m, 5H, H_ar_.); ^13^C-NMR (CDCl_3_) δ ppm 20.57, 20.89, 31.60 (5 × CH_2_, cyclohexyl), 25.62 (CH_3_), 42.66 (CH_2_-C_6_H_5_), 55.31 (O=C-CH_2_-N), 60.91 (Cq), 126.91, 127.61, 128.49, 128.68, 129.19 (CH_ar_.), 129.90 130.16, 145.41 (3 × C_ar_.), 170.99, 176.41 (2 × C=O); MS (EI) *m*/*z* (%): 362.3 ([M]^+^, 84), 91.1(100), 200.2 (93); Anal. Calcd. for C_23_H_26_N_2_O_2_ (362.46): C, 76.21%; H, 7.23%; N, 7.73%. Found: C, 76.31%; H, 7.19%; N, 7.75%.

1-(4-Methylphenyl)-4-phenethyl-1,4-diazaspiro[5.5]undecane-3,5-dione (**6r**). Yield: 59%; white solid, m.p. 92 °C; IR (KBr, *ν*, cm^−1^) absence of NH band at 3100 and exhibited bands at 1719, 1674 (imide carbonyls), 597, 559; ^1^H-NMR (CDCl_3_) δ ppm 1.48–1.94 (m, 10H, 5 × CH_2_, cyclohexyl), 2.25 (s, 3H, CH_3_), 2.87 (t, *J* = 7.7 Hz, 2H, CH_2_-C_6_H_5_), 4.04 (s, 2H, C*H_2_*-CH_2_-C_6_H_5_), 4.06 (s, 2H, O=C-CH_2_-N), 7.04 (d, 2H, *J* = 8.4 Hz, H_ar_.), 7.28 (d, 2H, *J* = 8.4 Hz, H_ar_.), 7.29–7.30 (m, 5H, H_ar_.); ^13^C-NMR (CDCl_3_) δ ppm 20.61, 20.91, 25.63 (5 × CH_2_, cyclohexyl), 31.58 (CH_3_), 34.12, 40.58 (CH_2_-C_6_H_5_, CH_2_-CH_2_-C_6_H_5_), 55.32 (O=C-CH_2_-NH_2_), 60.63 (Cq), 126.58, 126.72, 128.53, 129.10, 129.97 (CH_ar_.), 135.53, 138.44, 145.56 (3 × C_ar_.), 170.81, 176.58 (2 × C=O); MS (EI) *m*/*z* (%): 376.25 ([M]^+^, 70), 257.23 (100); Anal. Calcd. for C_24_H_28_N_2_O_2_ (376.49): C, 76.56%; H, 7.50%; N, 7.44%. Found: C, 76.33%; H, 7.75%; N, 7.29%. 

Methyl 2-(1-(4-methoxyphenyl)-3,5-dioxo-1,4-diazaspiro[5.5]undecan-4-yl)acetate (**6s**). Yield: (53.5%); yellow viscous oil; IR (KBr, *ν*, cm^−1^) absence of NH band at 3100 and exhibited bands at 1752 (carbonyl ester), 1626, 1675 (imide carbonyls), 619, 523; ^1^H-NMR (CDCl_3_) δ ppm 1.07–1.47 (m, 10H, 5 × CH_2_, cyclohexyl), 3.61 (s, 3H, COOCH_3_), 3.73 (s, 3H, OCH_3_), 4.22 (s, 2H, O=C-CH_2_-N), 4.64 (s, 2H, CH_2_-COOCH_3_), 6.75 (d, 2H, *J* = 8.6 Hz, H_ar_.), 7.04 (d, 2H, *J* = 8.6 Hz, H_ar_.); ^13^C-NMR (CDCl_3_) δ ppm 22.88, 25.51, 32.94 (5 × CH_2_, cyclohexyl), 37.70 (CH_2_COOCH_3_), 51.84, 52.10 (O=C-CH_2_-N, CH_2_COOCH_3_), 55.24 (OCH_3_), 67.64 (Cq), 113.38, 127.76 (CH_ar_.), 141.72, 156.29 (2 × C_ar_.), 168.2, 170.13, 178.70 (3 × C=O); MS (EI) *m*/*z* (%): 360 ([M]^+^, 0.5), 218.2 (100), 77 (5); Anal. Calcd. for C_19_H_24_N_2_O_5_ (360.40): C, 63.32%; H, 6.71%; N, 7.77%. Found: C, 63.33%; H, 6.81%; N, 7.91%. 

1-(4-Methoxyphenyl)-4-benzyl-1,4-diazaspiro[5.5]undecane-3,5-dione (**6t**). Yield: 63%; yellow viscous oil; IR (KBr, *ν*, cm^−1^) absence of NH band at 3100 and exhibited bands at 1725, 1675 (imide carbonyls), 615, 516; ^1^H-NMR (CDCl_3_) δ ppm 1.41-1.90 (m, 10H, 5 × CH_2_, cyclohexyl), 2.28 (s, 3H, OCH_3_), 4.16 (O=C-CH_2_-N), 4.94 (s, 2H, CH_2_-C_6_H_5_), 6.67 (d, 2H, *J* = 7.6 Hz, H_ar_.), 6.90 (d, 2H, *J* = 7.6 Hz, H_ar_.), 7.23–7.29 (m, 5H, H_ar_.); ^13^C-NMR (CDCl_3_) δ ppm 20.80, 25.59, 36.66 (5 × CH_2_, cyclohexyl), 42.66 (CH_2_-C_6_H_5_), 56.84, 59.07 (O=C-CH_2_-N, OCH_3_), 69.94 (Cq), 114.35, 124.97, 127.53, 128.46, 128.87 (CH_ar_.), 129.782, 136.89, 146.25 (3 × C_ar_.), 170.62, 176.18 (2 × C=O); MS (EI) *m*/*z* (%): 378.4 ([M]^+^, 7), 91.12 (100); Anal. Calcd. for C_23_H_26_N_2_O_3_ (378.46): C, 72.99%; H, 6.92%; N, 7.40%. Found: C, 72.75%; H, 6.78%; N, 7.52%.

1-(4-Methoxyphenyl)-4-phenethyl-1,4-diazaspiro[5.5]undecane-3,5-dione (**6u**). Yield: 66.5%; yellow viscous oil; IR (KBr, *ν*, cm^−1^) absence of NH band at 3100 and exhibit bands at 1725, 1685 (imide carbonyls), 671, 538; ^1^H-NMR (CDCl_3_) δ ppm 1.23–2.05 (m, 10H, 5 × CH_2_, cyclohexyl), 2.81 (s, 2H, CH_2_-C_6_H_5_), 3.72 (s, 3H, OCH_3_), 3.77(s, 2H, C*H_2_*-CH_2_-C_6_H_5_), 4.20 (s, 2H, O=C-CH_2_-N), 6.80–6.81 (m, 5H, H_ar_.), 7.13 (s, 4H, H_ar_.); ^13^C-NMR (CDCl_3_) δ ppm 22.78, 22.99, 32.11 (5 × CH_2_, cyclohexyl), 32.78, 38.02 (CH_2_-C_6_H_5_, CH_2_-CH_2_-C_6_H_5_), 55.52, 55.70 (O=C-CH_2_-N, OCH_3_), 68.20 (Cq), 113.66, 114.60, 117.64, 127.65, 128.23 (CH_ar_.), 138.23, 140.11, 157.74 (3 × C_ar_.), 170.22, 176.11 (2 × C=O); MS (EI) *m*/*z* (%): 392.39 ([M]^+^, 14), 105 (60); Anal. Calcd. for C_24_H_28_N_2_O_3_ (392.49): C, 73.44%; H, 7.19%; N, 7.14%. Found: C, 73.59%; H, 7.15%; N, 7.24%.

#### 3.1.7. General Procedure for the Synthesis of 2-(6-Aryl-8,10-dioxo-6,9-diazaspiro[4.5]decan-9-yl)acetamides (**7a**–**c**) and 2-(1-Aryl-3,5-dioxo-1,4-diazaspiro[5.5]undecan-4-yl)acetamides (**7d**–**f**)

Chloroacetamide (6.55 g, 0.07 mol) was added to a cold solution of the appropriate cyclized compound **5a**–**f** in acetone (100 mL) in the presence of K_2_CO_3_ (1.38 g, 0.01 mol) and a catalytic amount of tetrabutylammoniun bromide (0.32 g, 0.001 mol) as a phase transfer catalyst. The reaction mixture was heated under reflux for 7 h. The reaction mixture was filtered off and acetone was evaporated under reduced pressure to give compounds **7a**–**f**. The crude **7a**–**f** were purified *via* recrystallization from ethanol.

2-(8,10-Dioxo-6-phenyl-6,9-diazaspiro[4.5]decan-9-yl)acetamide (**7a**). Yield: 95%; yellowish white solid m.p. 104 °C; IR (KBr, *ν*, cm^−1^) exhibited bands at 3383.14, 3180.62 (NH_2_), 1674.21, 1647.21, 1614 (3 × C=O); ^1^H-NMR (CDCl_3_) δ ppm 1.75–1.96 (m, 6H, 3 × CH_2_, cyclopentyl), 2.35 (br.s, 2H, CH_2_-cyclopentyl), 4.05, 4.44 (2 × s, 4H, O=C-CH_2_-N, CH_2_-C=O), 5.97 (s, 2H, NH_2_), 6.51 (s, 3H, H_ar_.), 7.01–7.24 (m, 2H, H_ar_.); ^13^C-NMR (CDCl_3_) δ ppm 25.23, 36.73 (4 × CH_2_, cyclopentyl), 41.36 (CH_2_-C=O), 56.67 (O=C-CH_2_-N), 69.83 (Cq), 124.83, 129.30, 131.03 (CH_ar_.), 148.58 (C_ar_.), 169.08, 169.20, 176.02 (3 × C=O); MS (EI) *m*/*z* (%): 301.26 ([M]^+^, 7), 77.11 (100); Anal. Calcd. for C_16_H_19_N_3_O_3_ (301.34): C, 63.77%; H, 6.36%; N, 13.94%. Found: C, 63.79%; H, 6.35%; N, 13.92%.

2-(8,10-Dioxo-6-(4-methylphenyl)-6,9-diazaspiro[4.5]decan-9-yl)acetamide (**7b**). Yield: 98%; yellowish white solid m.p. 120 °C; IR (KBr, *ν*, cm^−1^) exhibited bands at 3383.14, 3197.98 (NH_2_), 1658.78, 1645.28, 1620.21 (3 × C=O); ^1^H-NMR (CDCl_3_) δ ppm 1.31-1.93 (m, 8H, 4 × CH_2_, cyclopentyl), 3.73 (s, 3H, CH_3_), 3.99, 4.48 (2s, 4H, O=C-CH_2_-N, CH_2_-C=O), 6.14 (s, 2H, NH_2_), 6.78 (d, 2H, *J* = 8.7 Hz, H_ar_.), 7.18 (d, 2H, *J* = 8.6 Hz, H_ar_.); ^13^C-NMR (CDCl_3_) δ ppm 20.55, 31.52 (4 × CH_2_, cyclopentyl), 25.51 (CH_3_), 41.37 (CH_2_-C=O), 55.45 (O=C-CH_2_-N), 61.26 (Cq), 114.48, 128.67 (CH_ar_.), 140.59, 157.7 (2 × C_ar_.), 169.56, 171.05, 176.44 (3 × C=O); MS (EI) *m*/*z* (%): 317.28 ([M + 2]^+^, 16), 121.16 (100); Anal. Calcd. for C_17_H_21_N_3_O_3_ (315.37): C, 64.74%; H, 6.71%; N, 13.32%. Found: C, 64.77%; H, 6.69%; N, 13.34%.

2-(8,10-Dioxo-6-(4-methoxyphenyl)-6,9-diazaspiro[4.5]decane-9-yl)acetamide (**7c**). Yield: 98%; yellowish white solid m.p. 73 °C; IR (KBr, *ν*, cm^−1^) exhibited bands at 3383.93, 3294.42 (NH_2_), 1658.78, 1639.49, 1616.35 (3 × C=O); ^1^H-NMR (CDCl_3_) δ ppm 1.02–1.47 (m, 4H, 2 × CH_2_, cyclopentyl), 1.68–2.26 (m, 4H, 2 × CH_2_, cyclopentyl), 3.77 (s, 3H, OCH_3_), 4.10, 4.13 (2 × s, 4H, O=C-CH_2_-N, CH_2_-C=O-N), 6.41 (s, 2H, NH_2_), 6.80 (d, 2H, *J* = 7.5 Hz, H_ar_.), 7.05 (d, 2H, *J* = 7.5 Hz, H_ar_.); ^13^C-NMR (CDCl_3_) δ ppm 23.87, 36.40 (4 × CH_2_, cyclopentyl), 42.05 (CH_2_-C=O), 55.66 (O=C-CH_2_-N, OCH_3_), 70.27 (Cq), 114.35, 126.88 (CH_ar_.), 141.71, 157.07 (2 × C_ar_.), 169.26, 169.29, 176.12 (3 × C=O); MS (EI) *m*/*z* (%): 331.3 ([M]^+^, 0.44), 67.17 (100); Anal. Calcd. for C_17_H_21_N_3_O_4_ (331.37): C, 67.62%; H, 6.39%; N, 12.68%. Found: C, 67.61%; H, 6.37%; N, 12.65%.

2-(3,5-Dioxo-1-phenyl-1,4-diazaspiro[5.5]undecan-4-yl)acetamide (**7d**). Yield: 97%; yellowish white solid m.p. 110 °C; IR (KBr, *ν*, cm^−1^) exhibited bands at 3385.07, 3188.3 (NH_2_), 1670, 1647.2, 1618.2 (3 × C=O); ^1^H-NMR (CDCl_3_) δ ppm 1.18–2.01 (m, 10H, 5 × CH_2_, cyclohexyl), 4.07, 4.47 (2 × s, 4H, O=C-CH_2_-N, CH_2_-C=O), 6.66 (s, 2H, NH_2_), 7.08-7.13 (m, 5H, H_ar_.); ^13^C-NMR (CDCl_3_) δ ppm of 24.03, 25.07, 33.15 (5 × CH_2_, cyclohexyl), 53.85, 54.93 (CH_2_-C=O, O=C-CH_2_-N), 58.92 (Cq), 127.34, 129.07, 129.3 (CH_ar_.), 147.97 (C_ar_.), 169.45, 169.78, 171.27 (3 × C=O); MS (EI*) m*/*z* (%): 315.26 ([M]^+^, 4.7), 58.17 (51), 100.2 (100); Anal. Calcd. for C_17_H_21_N_3_O_3_ (315.37): C, 64.74%; H, 6.71%; N, 13.32%. Found: C, 64.77%; H, 6.73%; N, 13.35%.

2-(3,5-Dioxo-1-(4-methylphenyl)-1,4-diazaspiro[5.5]undecan-4-yl)acetamide (**7e**). Yield: 94%; yellowish white solid m.p. 100 °C; IR (KBr, *ν*, cm^−1^) exhibited bands at 3456.14, 3383.14 (NH_2_), 1662.6, 1647.2, 1614.4 (3 × C=O); ^1^H-NMR (CDCl_3_) δ ppm of 1.32–1.57 (m, 10H, 5 × CH_2_, cyclohexyl), 2.18 (s, 3H, CH_3_) 3.94, 4.50 (2 × s, 4H, O=C-CH_2_-N, CH_2_-C=O), 5.97, 6.58 (2 × s, 2H, NH_2_), 6.98–7.29 (m, 4H, H_ar_.); ^13^C-NMR (CDCl_3_) δ ppm 20.35, 22.86, 23.49 (5 × CH_2_, cyclohexyl), 25.44 (CH_3_), 41.36 (CH_2_-C=O), 53.58 (O=C-CH_2_-N), 60.58 (Cq), 127.16, 135.49 (CH_ar_.), 129.41, 145.36 (2 × C_ar_.), 169.0, 169.50, 176.29 (3 × C=O); MS (EI) *m*/*z* (%): 329.32 ([M]^+^, 50), 257.28 (40), 100.16 (100), 142.18 (63); ); Anal. Calcd. for C_18_H_23_N_3_O_3_ (329.39): C, 65.63%; H, 7.04%; N, 12.76%. Found: C, 65.66%; H, 7.12%; N, 12.78%.

2-(3,5-Dioxo-1-(4-methoxyphenyl)-1,4-diazaspiro[5.5]undecan-4-yl)acetamide (**7f**). Yield: 97%; buff solid, m.p. 94 °C; IR (KBr, *ν*, cm^−1^) exhibited bands at 3383.14, 3186.4 (NH_2_), 1678.07, 1670.35, 1654.92 (3 × C=O); ^1^H-NMR (CDCl_3_) δ ppm 1.80–2.38 (m, 10H, 5 × CH_2_, cyclohexyl), 4.03 (s, 3H, OCH_3_), 4.28, 4.51 (2s, 4H, O=C-CH_2_-N, CH_2_-C=O), 7.01 (d, 2H, *J* = 8.4 Hz, H_ar_.), 7.08 (d, 2H, *J* = 8.4 Hz, H_ar_.) 7.26 (s, 2H, NH_2_); ^13^C-NMR (CDCl_3_) δ ppm 21.25, 25.19, 36.51 (5 × CH_2_, cyclohexyl), 41.50, 42.14 (CH_2_-C=O, O=C-CH_2_-N), 56.39 (OCH_3_), 70.43 (Cq), 124.64, 129.81 (CH_ar_.), 135.25, 145.33 (2 × C_ar_.), 169.04, 169.76, 175.66 (3 × C=O); MS (EI) *m*/*z* (%): 315.28 ([M-OCH_3_]^+^, 13), 105.14 (100), 287.31(10); Anal. Calcd. for C_18_H_23_N_3_O_4_ (345.39): C, 62.59%; H, 6.71%; N, 12.17%. Found: C, 62.57%; H, 6.74%; N, 12.19%.

#### 3.1.8. General Procedure for the Synthesis of 2-(6-Aryl-8,10-dioxo-6-phenyl-6,9-diazaspiro[4.5]decan-9-yl)acetonitriles (**8a**–**c**) and 2-(1-Aryl-3,5-dioxo-1,4-diazaspiro[5.5]undecan-4-yl)acetonitriles (**8d**–**f**)

Trifluroacetic anhydride (6.61 g, 0.03 mol) was added to a solution of the appropriate amide **7a**–**f** (0.02 mol) in THF (40 mL) at 0–5 °C. The reaction mixture was stirred at room temperature for 2 h (monitored by TLC). Ammonium bicarbonate (12.43 g, 0.16 mol) was added portion-wise during 5–10 min. and the reaction mixture was stirred at room temperature for a further 45 min., concentrated under vacuum, washed with water (2 × 20 mL) and extracted with ethyl acetate (3 × 30 mL). The organic layer was dried (Na_2_SO_4_) and evaporated under reduced pressure to afford compounds **8a**–**f**. The crude compounds **8a**–**f** were purified using column chromatography (chloroform:ethyl acetate, 9:1).

2-(8,10-Dioxo-6-phenyl-6,9-diazaspiro[4.5]decan-9-yl)acetonitrile (**8a**). Yield: 97%; yellowish white solid m.p.70 °C; IR (KBr, *ν*, cm^−1^) absence of amidic NH_2_ and exhibited bands at 2848.86 (CN), 1743.65, 1687.71 (2 × C=O); ^1^H-NMR (CDCl_3_) δ ppm 1.20–2.30 (m, 8H, 4 × CH_2_, cyclopentyl), 4.25, 4.64 (2 × s, 4H, O=C-CH_2_-N and CH_2_-CN), 6.68–7.27 (m, 5H, H_ar_.); ^13^C-NMR (CDCl_3_) δ ppm 25.18, 26.33 (4 × CH_2_, cyclopentyl), 29.54 (CH_2_-CN), 56.61 (O=C-CH_2_-N), 69.70 (Cq), 114.36 (CN), 124.60, 124.91, 129.27 (CH_ar_.), 148.29 (C_ar_.), 169.36, 175.26 (2 × C=O); MS (EI) *m*/*z* (%): 283.25 ([M]^+^, 25), 91.09 (100), 243.23 (15); Anal. Calcd. for C_16_H_17_N_3_O_2_ (283.33): C, 67.83%; H, 6.05%; N, 14.83%. Found: C, 67.85%; H, 6.15%; N, 14.82%.

2-(8,10-Dioxo-6-(4-methylphenyl)-6,9-diazaspiro[4.5]decan-9-yl)acetonitrile (**8b**). Yield: 90%; yellowish white solid m.p. 82 °C; IR (KBr, *ν*, cm^−1^) absence of amidic NH_2_ and exhibited bands at 2226.71 (CN), 1735.93, 1689.64 (2 × C=O); ^1^H-NMR (CDCl_3_) δ ppm 1.26–1.79 (m, 8H, 4 × CH_2_, cyclopentyl), 2.34 (s, 3H, CH_3_), 4.09 (s, 2H, O=C-CH_2_-N), 4.71 (s, 2H, CH_2_-CN), 6.80 (d, 2H, *J* = 8.6 Hz, H_ar_.), 6.90 (d, 2H, *J* = 8.6 Hz, H_ar_.); ^13^C-NMR (CDCl_3_) δ ppm 20.51, 29.69 (2 × CH_2_, cyclopentyl), 26.26 (CH_3_), 29.54 (CH_2_-CN), 54.87 (O=C-CH_2_-N), 61.33 (Cq), 114.46 (CN), 127.97, 128.69 (CH_ar_.), 140.16, 157.65 (2 × C_ar_.), 170.66, 175.48 (2 × C=O); MS (EI) *m*/*z* (%): 299.26 ([M + 2]^+^, 16), 121.15 (100); Anal. Calcd. for C_17_H_19_N_3_O_2_ (297.35): C, 68.67%; H, 6.44%; N, 14.13%. Found: C, 68.68%; H, 6.42%; N, 14.15%. 

2-(8,10-Dioxo-6-(4-methoxyphenyl)-6,9-diazaspiro[4.5]decane-9-yl)acetonitrile (**8c**). Yield: 77%; yellow viscous oil; IR (KBr, *ν*, cm^−1^) absence of amidic NH_2_ and exhibited bands at 2310.70 (CN), 1743.65, 1629.85 (2 × C=O); ^1^H-NMR (CDCl_3_) δ ppm 1.27–1.55 (m, 4H, 2 × CH_2_, cyclopentyl), 1.79–2.21 (m, 4H, 2 × CH_2_, cyclopentyl), 3.77 (s, 3H, OCH_3_), 4.13 (s, 2H, O=C-CH_2_-N), 4.78 (s, 2H, CH_2_-CN), 6.89 (d, 2H, *J* = 7.5 Hz, H_ar_.), 6.97 (d, 2H, *J* = 7.5 Hz, H_ar_.); ^13^C-NMR (CDCl_3_) δ ppm 26.19, 38.30 (2 × CH_2_, cyclopentyl), 29.06 (CH_2_-CN), 55.44 (O=C-CH_2_-N), 56.16 (OCH_3_), 65.11 (Cq), 114.62 (CN), 127.98, 131.32 (CH_ar_.), 142.62, 157.30 (2 × C_ar_.), 169.54, 175.16 (2 × C=O); MS (EI) *m*/*z* (%): 312.43 ([M − 1]^+^, 4), 57.15 (100); Anal. Calcd. for C_17_H_19_N_3_O_3_ (313.35): C, 65.16%; H, 6.11%; N, 13.41%. Found: C, 65.14%; H, 6.13%; N, 13.42%.

2-(3,5-Dioxo-1-phenyl-1,4-diazaspiro[5.5]undecan-4-yl)acetonitrile (**8d**). Yield: 90%; yellow viscous oil; IR (KBr, *ν*, cm^−1^) absence of amidic NH_2_ and exhibited bands at 2254.79 (CN), 1735.9, 1705.07 (2 × C=O); ^1^H-NMR (CDCl_3_) δ ppm 1.18 (s, 4H, 2 × CH_2_, cyclohexyl), 1.45–1.79 (m, 6H, 3 × CH_2_, cyclohexyl), 4.09 (s, 2H, O=C-CH_2_-N), 4.69 (s, 2H, CH_2_-CN), 6.93-7.24 (m, 5H, H_ar_.); ^13^C-NMR (CDCl_3_) δ ppm 25.01, 29.07, 29.25 (5 × CH_2_, cyclohexyl), 29.44 (CH_2_-CN), 54.22 (O=C-CH_2_-N), 60.85 (Cq), 114.29 (CN), 128.95, 129.33, 129.64 (CH_ar_.), 147.36 (C_ar_.), 169.76, 175.30 (2 × C=O); MS (EI) *m*/*z* (%): 297.28 ([M]^+^, 6), 257.2 (4), 77.14 (100); Anal. Calcd. for C_17_H_19_N_3_O_2_ (297.35): C, 68.67%; H, 6.44%; N, 14.13%. Found: C, 68.69%; H, 6.46%; N, 14.11%.

2-(3,5-Dioxo-1-(4-methylphenyl)-1,4-diazaspiro[5.5]undecan-4-yl)acetonitrile (**8e**). Yield: 94.5%; yellowish white solid m.p. 120–122 °C; IR (KBr, *ν*, cm^−1^) absence of amidic NH_2_ and exhibited bands at 1888.31 (CN), 1741.72, 1989.6 (2 × C=O); ^1^H-NMR (CDCl_3_) δ ppm 1.28 (s, 2H, CH_2_, cyclohexyl), 1.45–2.01 (m, 8H, 4 × CH_2_, cyclohexyl), 2.32 (s, 3H, CH_3_), 4.16 (s, 2H, O=C-CH_2_-N ), 4.74 (s, 2H, CH_2_-CN), 6.91 (d, 2H, *J* = 7.0 Hz, H_ar_.), 7.15 (d, 2H, *J* = 7.0 Hz, H_ar_.); ^13^C-NMR (CDCl_3_) δ ppm 22.70 (CH_3_), 23.06, 24.76, 25.36 (5 × CH_2_, cyclohexyl), 31.33 (CH_2_-CN), 54.92 (O=C-CH_2_-N), 60.98 (Cq), 114.23 (CN), 129.77, 130.24 (CH_ar_.), 136.09, 144.74 (2 × C_ar_.), 169.89, 175.37 (2 × C=O); MS (EI) *m*/*z* (%): 311.27 ([M]^+^, 16), 91.15 (100); Anal. Calcd. for C_18_H_21_N_3_O_2_ (311.38): C, 69.43%; H, 6.80%; N, 13.49%. Found: C, 69.44%; H, 6.81%; N, 13.47%.

2-(3,5-Dioxo-1-(4-methoxyphenyl)-1,4-diazaspiro[5.5]undecan-4-yl)acetonitrile (**8f**). Yield: 75%; brown viscous oil; IR (KBr, *ν*, cm^−1^) absence of amidic NH_2_ and exhibited bands at 2260.57 (CN), 1687.71, 1676.14 (2 × C=O); ^1^H-NMR (CDCl_3_) δ ppm 1.76–2.06 (m, 10H, 5 × CH_2_, cyclohexyl), 3.75 (s, 3H, OCH_3_), 4.27 (s, 2H, O=C-CH_2_-N), 4.69 (s, 2H, CH_2_-CN), 6.91 (d, 2H, *J* = 7.8 Hzs, H_ar_.), 7.04 (d, 2H, *J* = 7.8 Hz, H_ar_.); ^13^C-NMR (CDCl_3_) δ ppm 20.41, 26.23, 31.40 (5 × CH_2_, cyclohexyl), 29.75 (CH_2_-CN), 54.86 (O=C-CH_2_-N), 61.37 (OCH_3_) 68.26 (Cq), 114.72 (CN), 127.82, 128.09 (CH_ar_.), 140.19, 157.88 (2 × C_ar_.), 170.13, 175.43 (2 × C=O); MS (EI) *m*/*z* (%): 327.23 ([M]^+^, 85), 121.11 (100), 287.22 (20); Anal. Calcd. for C_18_H_21_N_3_O_3_ (327.38): C, 66.04%; H, 6.47%; N, 12.84%. Found: C, 66.13%; H, 6.49%; N, 12.85%.

#### 3.1.9. General Procedure for the Synthesis of 6-Aryl-9-(1*H*-tetrazol-5-yl)methyl)-6,9-diazaspiro[4.5] decane-8,10-diones (**6j**–**l**) and 1-Aryl-4-((1*H*-tetrazol-5-yl)methyl)-1,4-diazaspiro[5.5]undecane-3,5-diones (**6v**–**x**)

Anhydrous AlCl_3_ (13.3 g, 0.1 mol) was added to a cold dry THF (200 mL) under stirring during 10 min. Thereafter, NaN_3_ (28.9 g, 0.45 mol) was added portion-wise through 10 min. The appropriate penultimate nitrile derivative **8a**–**f** was added and the reaction mixture was stirred under refluxed for 24 h. After cooling, the reaction mixture was filtered and the filtrate was evaporated under vacuum. The crude residues were purified through column chromatography (chloroform:ethyl acetate, 9:1) to give the ultimate respective compounds **6j**–**l** and **6v**–**x**.

6-Phenyl-9-((1*H*-tetrazol-5-yl)methyl)-6,9-diazaspiro[4.5]decane-8,10-dione (**6j**). Yield: 71%; colorless viscous oil; IR (KBr, *ν*, cm^−1^) exhibited band at 3419 (NH) and disappearance of CN band; ^1^H-NMR (CDCl_3_) δ ppm 1.11–2.23 (m, 8H, 4 × CH_2_, cyclopentyl), 4.22 (s, 2H, O=C-CH_2_-N), 5.14 (s, 2H, N-CH_2_-tetrazole)), 6.75–7.07 (m, 5H, H_ar_.), 7.37 (s, 1H, NH); ^13^C-NMR (CDCl_3_) δ ppm 25.15, 29.77 (4 × CH_2_, cyclopentyl), 38.86 (N-CH_2_-tetrazole), 56.70 (O=C-CH_2_-N), 69.98 (Cq), 124.92, 129.42, 132.50 (CH_ar_.), 148.48 (C_ar_.), 153.55 (C=N-tetrazole), 170.55, 176.06 (2 × C=O); MS (EI) *m*/*z* (%): 326.43 ([M]^+^, 33), 327.27 (100); Anal. Calcd. for C_16_H_18_N_6_O_2_ (326.35): C, 58.88%; H, 5.56%; N, 25.75%. Found: C, 58.66%; H, 5.51%; N, 25.72%. 

6-(4-Methylphenyl)-9-((1*H*-tetrazol-5-yl)methyl)-6,9-diazaspiro[4.5]decane-8,10-dione (**6k**). Yield: 61%; yellow viscous oil; IR (KBr, *ν*, cm^−1^) exhibited band at 3419 (NH) and disappearance of CN band; ^1^H-NMR (CDCl_3_) δ ppm 1.47 (s, 3H, CH_3_), 1.51–1.56 (m, 4H, 2 × CH_2_, cyclopentyl), 1.77 (br.s, 4H, 2 × CH_2_ cyclopentyl), 3.91 (s, 2H, O=C-CH_2_-N), 4.10 (s, 2H, N-CH_2_-tetrazole), 5.21 (s, 1H, NH), 6.83 (d, 2H, *J* = 6.0 Hz, H_ar_.), 7.12 (d, 2H, *J* = 6.0 Hz, H_ar_.); ^13^C-NMR (CDCl_3_) δ ppm 25.38 (CH_3_), 30.02, 34.84 (4 × CH_2_, cyclopentyl), 50.98 (N-CH_2_-tetrazole), 55.59 (O=C-CH_2_-N), 60.89 (Cq), 125.36, 128.92 (CH_ar_.), 140.92, 151.94 (2 × C_ar_.), 157.55(C=N-tetrazole), 168.93, 170.71 (2 × C=O); MS (EI) *m*/*z* (%): 340.29 ([M]^+^, 0.5), 121.14 (100); Anal. Calcd. for C_17_H_20_N_6_O_2_ (340.38): C, 59.99%; H, 5.92%; N, 24.69%. Found: C, 59.74%; H, 5.82%; N, 24.67%.

6-(4-Methoxyphenyl)-9-((1*H*-tetrazol-5-yl)methyl)-6,9-diazaspiro[4.5]decane-8,10-dione (**6l**). Yield: 63.3%; yellow viscous oil; IR (KBr, *ν*, cm^−1^) exhibited band at 3421.72 (NH) and disappearance of CN band; ^1^H-NMR (CDCl_3_) δ ppm 1.47 (br.s, 6H, 3 × CH_2_, cyclopentyl), 2.19 (br.s, 2H, CH_2_ cyclopentyl), 3.73 (s, 3H, OCH_3_), 3.80 (s, 2H, O=C-CH_2_-N), 4.21 (s, 2H, N-CH_2_-tetrazole)), 6.64 (s, 1H, NH), 6.99 (d, 2H, *J* = 6.0 Hz, H_ar_.), 7.25 (d, 2H, *J* = 6.0 Hz, H_ar_.); ^13^C-NMR (CDCl_3_) δ ppm 23.08, 30.43 (4 × CH_2_, cyclopentyl), 51.01 (N-CH_2_-tetrazole), 55.83 (O=C-CH_2_-N), 59.45 (OCH_3_), 61.02 (Cq), 128.49, 132.02 (CH_ar_.), 140.0, 152.0 (2 × C_ar_.), 159.96 (C=N-tetrazole), 160.01, 169.1 (2 × C=O); Anal. Calcd. for C_17_H_20_N_6_O_3_ (356.38): C, 57.29%; H, 5.66%; N, 23.58%. Found: C, 57.11%; H, 5.64%; N, 23.38%.

1-Phenyl-4-((1*H*-tetrazol-5-yl)methyl)-1,4-diazaspiro[5.5]undecane-3,5-dione (**6v**). Yield: 75%; yellow viscous oil; IR (KBr, *ν*, cm^−1^) exhibited band at 3400 (NH) and disappearance of CN band; ^1^H-NMR (CDCl_3_) δ ppm 0.87 (br.s, 2H, CH_2_ cyclohexyl), 1.18–1.98 (m, 8H, 4 × CH_2_, cyclohexyl), 4.09 (s, 2H, O=C-CH_2_-N), 5.03 (s, 2H, N-CH_2_-tetrazole), 7.05–7.13 (m, 5H, H_ar_ and 1H, NH); ^13^C-NMR (CDCl_3_) δ ppm 13.58, 25.37, 31.36 (5 × CH_2_, cyclohexyl), 55.01 (N-CH_2_-tetrazole), 58.88 (O=C-CH_2_-N), 60.77 (Cq), 125.93, 127.15, 129.38 (CH_ar_.), 147.86 (C_ar_.), 153.74 (C=N-tetrazole), 170.57, 175.98 (2 × C=O); MS (EI) *m*/*z* (%): 340.29 ([M]^+^, 3), 77.13 (100); Anal. Calcd. for C_17_H_20_N_6_O_2_ (340.38): C, 59.99%; H, 5.92%; N, 24.69%. Found: C, 59.78%; H, 5.91%; N, 24.68%.

1-(4-Methylphenyl)-4-((1*H*-tetrazol-5-yl)methyl)-1,4-diazaspiro[5.5]undecane-3,5-dione (**6w**). Yield: 61%; yellow viscous oil; IR (KBr, *ν*, cm^−1^) exhibited band at 3419.79 (NH) and disappearance of CN band; ^1^H-NMR (CDCl_3_) δ ppm 1.47 (s, 3H, CH_3_), 1.51–1.59 (m, 6H, 3 × CH_2_, cyclohexyl), 2.14 (br.s, 4H, 2 × CH_2_ cyclohexyl), 4.05 (s, 2H, O=C-CH_2_-N ), 4.30 (s, 2H, N-CH_2_-tetrazole), 6.65 (s, 1H, NH), 7.16 (d, 2H, *J* = 6.0 Hz, H_ar_.), 7.19 (d, 2H, *J* = 6.0 Hz, H_ar_.); ^13^C-NMR (CDCl_3_) δ ppm 22.66, 31.50, 34.94 (5 × CH_2_, cyclohexyl), 29.74 (CH_3_), 45.801 (N-CH_2_-tetrazole), 50.95 (O=C-CH_2_-N), 60.73 (Cq), 127.83, 130.13 (CH_ar_.), 140.10, 145.92 (2 × C_ar_.), 151.94 (C=N-tetrazole), 168.82, 170.42 (2 × C=O); MS (EI) *m*/*z* (%): 354.7 ([M]^+^, 3), 105.1 (100); Anal. Calcd. for C_18_H_22_N_6_O_2_ (354.41): C, 61.00%; H, 6.26%; N, 23.71%. Found: C, 61.15%; H, 6.22%; N, 23.61%.

1-(4-Methoxyphenyl)-4-((1*H*-tetrazol-5-yl)methyl)-1,4-diazaspiro[5.5]undecane-3,5-dione (**6x**). Yield: 66%; buff solid, m.p 73 °C; IR (KBr, *ν*, cm^−1^) exhibited band at 3419 (NH) and disappearance of CN band; ^1^H-NMR (CDCl_3_) δ ppm 1.18–1.88 (m, 10H, 5 × CH_2_, cyclohexyl), 3.75 (OCH_3_), 4.20 (s, 2H, O=C-CH_2_-N), 5.29 (s, 2H, N-CH_2_-tetrazole), 6.73 (d, 2H, *J* = 7.0 Hz, H_ar_.), 6.95 (d, 2H, *J* = 7.0 Hz, H_ar_.), 7.19 (s, 1H, NH); ^13^C-NMR (CDCl_3_) δ ppm 20.71, 25.04, 32.60 (5 × CH_2_, cyclohexyl), 36.59 (N-CH_2_-tetrazole), 41.32 (O=C-CH_2_-N), 56.50 (OCH_3_), 70.07 (Cq), 127.79, 129.88 (CH_ar_.), 134.86, 145.77 (2 × C_ar_.), 153.48 (C=N-tetrazole), 170.73, 176.09 (2 × C=O); MS (EI) *m*/*z* (%): 340.3 ([M-OCH_3_]^+^, 5), 105.17 (100); Anal. Calcd. for C_18_H_22_N_6_O_3_ (370.41): C, 58.37%; H, 5.99%; N, 22.69%. Found: C, 58.32%; H, 5.79%; N, 22.54%.

### 3.2. Anticonvulsant Activity

#### 3.2.1. Materials

**Animals**: The anticonvulsant activity of the target compounds **6a**–**x** was tested on Swiss strain adult male albino mice weighing 19–25 g. Animals were obtained from the Animals House Colony of the National Research Centre, Cairo, Egypt. Animals were housed in polypropylene cages under the standard conditions of light (12 h light/dark cycle) and temperature (23 ± 2 °C), and were allowed free access to water and maintained on a daily standard schedule of laboratory diet. Procedures involving animals and their care were performed after the Ethics Committee of the National Research Centre and in accordance with the recommendations for the proper care and use of laboratory animals, “Canadian Council on Animal Care Guidelines, 1984”. Additionally, all efforts were made to minimize animals suffering and to use only the number of animals necessary to produce reliable data.

**Drugs and Chemicals**: Phenobarbital (Memphis Co. for Pharm & Chem. Ind., Cairo, Egypt), Ethosuximide (Pfizer Co., Giza, Egypt), Diphenylhydantoin (Nasr Co., Giza, Egypt), Tween 80 and Pentylenetetrazole (Sigma, St. Loius, MO, USA) were used. Ethosuximide, Phenobarbital and Pentylenetetrazole (PTZ) were dissolved in physiologic saline solution, Diphenylhydantoin was dissolved in saline that was alkalinized slightly with 0.1 mmol potassium hydroxide. Reference drugs and tested compounds were administered intraperitoneally (i.p) in volumes of 0.1 mL/10 g of mice body weight.

#### 3.2.2. Methods

After 7 days of adaptation to laboratory conditions, the animals were randomly assigned to control, reference and tested experimental groups consisting of 6 mice. Each mouse was used only once and all tests were performed between 09:00 a.m. and 04:00 p.m. All the tested compounds were suspended in 7% Tween 80 as a vehicle.

**Subcutaneous Pentyleneteterazole (scPTZ)-induced Seizures Test** [28]: A PTZ dose of 85 mg/kg administered subcutaneously to mice causes seizures in more than 97% of the animals. This is called the convulsive dose 97 (CD_97_). The control experiments were performed using the solvent alone. The other groups each received individually the reference drugs Ethosuximide (150 mg/kg ≡ 1.06 mmol/kg) [29] and/or Phenobarbital (30 mg/kg ≡ 0.13 mmol/kg) [30] or one of the test compounds in graded doses, **6a**–**l** (1.5–50 mg/kg), **6m**–**x** (6–100 mg/kg). Thirty minutes later Pentylenetetrazole was administered subcutaneously in a loose fold of skin on the back of the neck in a dose of 85 mg/kg. Each animal was observed for 30 min after PTZ administration, failure to observe even a threshold seizure (a single episode of clonic spasms of at least 5 s duration) was defined as protection [31]

**Maximal Electroshock Seizure (MES) Test** [32]: Animals were randomly assigned to groups of 6 mice each. The first group served as the control group. The second group received Diphenylhydantoin (45 mg/kg) as a reference drug and the other groups of animals received the test compounds individually by intraperitoneal injection with the dose which induces 100% protection in the Pentylenetetrazole test. Thirty minutes later electroconvulsions were induced by a current (fixed current intensity of 25 mA, 0.2 s stimulus duration) delivered *via* ear-clip electrodes by a Rodent Shocker generator (constant-current stimulator Type 221, Hugo Sachs Elektronik, Freiburg, Germany). 

The maximal seizures typically consist of a short period of initial tonic flexion and a prolonged period of tonic extension (especially of the hind limbs) followed by terminal clonus. The typical seizure lasts approximately 22 s. Failure to extend the hind limbs to an angle with trunk greater than 90° is defined as protection [33].

**Neurotoxicity** [34]: This test is designed to detect minimal neurological deficit. In this test, the animals were trained to maintain equilibrium on a rotating 1-inch-diameter knurled plastic rod at a speed of 6 rev/min for at least 1 min in each of three trials using a rotarod device (UGO Basile, 47600, Varese, Italy). Only animals that fulfill this criterion were included in the experiment. The selected trained animals were classified into control and experimental groups. The animals in the experimental groups were given the reference drug or one of the test compounds via i.p. route at doses which exerted 100% protection in the PTZ test; meanwhile, the control group received the vehicle. Thirty minutes later, the mice were placed again on the rotating rod and the neurotoxicity was indicated by the inability of the animal to maintain equilibrium on the rod for at least 1 min.

#### 3.2.3. Determination of the ED_50_

Anticonvulsant activity of the test compounds was expressed in term of median effective dose (ED_50_) that is, the dose of drug required to produce the required biological response in 50% of animals. For determination of the ED_50_, groups of 8 mice were given a range of i.p. doses of the test compound until at least three points were established in the range of 15%–84% seizure protection. From the plot of these data, the respective ED_50_ value and the confidence limits were calculated [18].

## 4. Conclusions

The anticonvulsant potential of certain new 6-aryl-9-substituted-6,9-diazaspiro[4.5]decane-8,10-diones (**6a**–**l**) and 1-aryl-4-substituted-1,4-diazaspiro[5.5]undecane-3,5-diones (**6m**–**x**) was described. The title compounds **6a**–**x** showed good anticonvulsant activity especially in the scPTZ screen. Compound **6g** displayed an ED_50_ of 0.0043 mmol/kg in the scPTZ screen being about 14 and 214 fold more potent than the reference drugs, Phenobarbital and Ethosuximide, respectively. Compound **6e** exhibited an ED_50_ of 0.019 mmol/kg being about 1.8 fold more potent than that of the reference drug, Diphenylhydantoin in the MES screen. None of the test compounds exhibited any minimal motor impairment at the maximum administered dose in the neurotoxicity screen. 
